# Liquid Biopsy Is a Promising Tool for Genetic Testing in Idiopathic Pulmonary Fibrosis

**DOI:** 10.3390/diagnostics11071202

**Published:** 2021-07-02

**Authors:** Pierlorenzo Pallante, Umberto Malapelle, Mariantonia Nacchio, Roberta Sgariglia, Domenico Galati, Ludovica Capitelli, Serena Zanotta, Mario Galgani, Erica Piemonte, Alessandro Sanduzzi Zamparelli, Gaetano Rea, Marialuisa Bocchino

**Affiliations:** 1 Istituto per l’Endocrinologia e l’Oncologia Sperimentale (IEOS) “G. Salvatore”, Consiglio Nazionale delle Ricerche (CNR), 80131 Napoli, Italy; pallante@ieos.cnr.it; 2 Dipartimento di Sanità Pubblica, Università Federico II, 80131 Napoli, Italy; umberto.malapelle@unina.it (U.M.); mariantonia.nacchio@unina.it (M.N.); roberta.sgariglia@unina.it (R.S.); 3 Dipartimento di Onco-Ematologia e Diagnostica Innovativa, Istituto Nazionale Tumori-IRCCS-Fondazione “G. Pascale”, 80131 Napoli, Italy; d.galati@istitutotumori.na.it (D.G.); s.zanotta@istitutotumori.na.it (S.Z.); 4 Dipartimento di Medicina Clinica e Chirurgia, Università Federico II, 80131 Napoli, Italy; ludovica.capitelli@gmail.com (L.C.); alessandro.sanduzzi@unina.it (A.S.Z.); 5 Dipartimento di Medicina Molecolare e Biotecnologie Mediche, Università Federico II, 80131 Napoli, Italy; mario.galgani@unina.it (M.G.); erica.piemonte@unina.it (E.P.); 6 Dipartimento di Radiologia, Azienda Ospedaliera dei Colli, Ospedale Monaldi, 80131 Napoli, Italy; gaetano.rea@ospedalemonaldi.it

**Keywords:** idiopathic pulmonary fibrosis, liquid biopsy, cell-free DNA, MUC5B, genetics

## Abstract

Liquid biopsy, which allows the isolation of circulating cell-free (ccf) DNA from blood, is an emerging noninvasive tool widely used in oncology for diagnostic and prognosis purposes. Previous data have shown that serum cfDNA discriminates idiopathic pulmonary fibrosis (IPF) from other interstitial lung diseases. Our study aimed to measure plasma levels of ccfDNA in 59 consecutive therapy-naive and clinically stable IPF patients. The single nucleotide polymorphism (SNP) of the MUC5B gene promoter (rs35705950), associated with increased susceptibility of developing IPF, has been sought in plasma cfDNA and genomic DNA for comparison. Thirty-five age- and sex-matched healthy volunteers were recruited as the control group. Our results show that concentrations of small-size ccfDNA fragments were significantly higher in IPF patients than in controls and inversely correlated with lung function deterioration. Moreover, the median level of 104 ng/mL allowed discriminating patients with mild disease from those more advanced. The rs35705950 polymorphism was found in 11.8% of IPF patients and 8% of controls, with no differences. Complete concordance between ccfDNA and genomic DNA was detected in all control samples, while four out of seven IPF cases (57%) carrying the rs35705950 polymorphism were discordant from genomic DNA (7% of total IPF). Liquid biopsy is a suitable tool with optimistic expectations of application in the field of IPF. In analogy with cancer biology, finding some discrepancies between ccfDNA and genomic DNA in IPF patients suggests that the former may convey specific genetic information present in the primary site of the disease.

## 1. Introduction

Idiopathic pulmonary fibrosis (IPF) is a progressive/irreversible and poor prognosis entity of unknown origin whose incidence has increased in the last decades due to facilitated disease awareness. IPF is a specific fibrotic interstitial lung disease (ILD) associated with a histology and chest high resolution computed tomography (HRCT) pattern of usual interstitial pneumonia (UIP). Clinical behavior is heterogeneous, ranging from slow evolving to quick and sudden lung deterioration, thus challenging disease management [1,2]. Mortality is very high within the first five years from diagnosis in untreated patients, with survival rates (not exceeding 20–25%) close to those of lung and pancreas carcinomas [3,4]. Aging and cigarette smoke are the main risk factors for IPF. Environmental, occupational exposure, and a wide variety of genetic and epigenetic alterations are also involved. Since the first description in 2000 of disease-specific associated gene variants of surfactant proteins, both common and rare variants have been reported in multiple genes related to senescence, immunity, and inflammation [5]. Among the common variants (minor allele frequency >5%), the most studied is the single nucleotide polymorphism (SNP) in the promoter region of the mucin 5B (MUC5B) gene, rs35705950. Minor T allele, compared to G allele, is strongly associated with increased susceptibility to familial interstitial pneumonia and sporadic IPF [6,7]. Of interest, this polymorphism is specific to the CT UIP pattern in the setting of fibrotic idiopathic interstitial pneumonia [8]. Moreover, it has been related to a slower disease progression and improved survival in IPF patients [9].

Liquid biopsy, also known as fluid biopsy, is the noninvasive sampling and analysis of non-solid biological tissues, primarily blood. The concept of liquid biopsy was introduced more than 10 years ago for cancer biomarker discovery through the isolation of circulating tumor cells (CTS), circulating cell-free (ccf) DNA, extra-cellular vesicles, and other tumor-derived products (i.e, noncoding and messanger cell free RNA) [10]. Specifically, ccfDNA contains DNA fragments released from the tumor cells (circulating tumor DNA, ctDNA), thus carrying disease-specific genetic and epigenetic information. Recent ccfDNA-based studies have provided promising results with potential clinical application in early cancer detection, diagnosis, and prognosis [11,12]. IPF notably shares many similarities with lung cancer, ranging from genetics to clinical behavior [13]. Moreover, IPF itself is associated with a 10-fold increased risk of developing lung cancer [14]. In 2010, Casoni et al. first suggested that serum cfDNA may help discriminate patients affected by IPF from those with other ILDs [15]. To the best of our knowledge, no further efforts followed.

Based on this background, we asked whether circulating cfDNA may carry any disease-specific genetic information in a real-life cohort of newly diagnosed and therapy-naïve IPF patients. As a preliminary step, we first analyzed: a) The plasma concentrations and distribution profile of short-length cfDNA fragments, and b) the relationship of plasma cfDNA with lung function and disease severity. Then, to address the main study aim, we investigated the MUC5B promoter polymorphism rs35705950 at the ccfDNA level in comparison to genomic DNA to discriminate germline from disease-acquired mutations. 

## 2. Materials and Methods

### 2.1. Study Population

Fifty-nine consecutive patients with newly diagnosed and therapy-naïve IPF referring to our clinic from January 2018 to January 2020 were enrolled. IPF diagnosis was made according to the 2018 official diagnostic guidelines [2]. Exclusion criteria were related to the diagnosis of respiratory diseases other than IPF, acute exacerbation in the four weeks before the study visit, and lung cancer coexistence. Thirty-five age- and sex-comparable volunteers with no lung disease, including patient relatives or subjects referring to us for lung function screening, were enrolled as the control group. The study was retrospectively conducted following the amended Declaration of Helsinki and was approved by the local Ethics committee (Registration number: 97/2020). Enrolled patients gave their written informed consent to participate in the study, and all data of interest were collected anonymously from clinical charts into a dedicated database. Spirometry, lung volume measurement, and determination of the hemoglobin (Hb)-adjusted single-breath diffusing lung capacity of the carbon monoxide (DLCO_sb_) were performed using a computer-assisted spirometer (Quark PFT 2008 Suite Version Cosmed Ltd., Rome, Italy) according to international standards [16,17,18]. The 6-min walk test (6-MWT) was performed by trained hospital staff according to guidelines in those patients with a basal peripheral oxygen saturation >90% in ambient air [19]. The GAP (stages I, II, and III) and TORVAN (stages I, II, III, and IV) disease scores were recorded as previously described [20,21].

### 2.2. Extraction and Quantification of Circulating Cell-Free DNA

Circulating cell-free DNA was extracted from 1 mL of plasma samples, previously stored at −80 °C, with the QIAamp^®^ MinElute ccfDNA Mini Kit (Qiagen, Venlo, Netherlands) according to the manufacturer’s instructions. Briefly, the extraction protocol allows a pre-concentration of circulating nucleic acids onto magnetic beads and a subsequent purification of the resulting pre-eluate (40×) on columns. The procedure helps eliminate sample-to-sample contamination, and cleaned-up nucleic acids are free of proteins, nucleases, and other impurities. Quantification of ccfDNA was then measured with the Tape Station 2200 system (Agilent, Santa Clara, CA, USA) which automatically generates sample concentration and size. For each run, 1 µL of the ccfDNA sample was used. Concentration was expressed as ng/µL. Plasma concentration was obtained with the following formula: [(ng/μL) × 40]/mL.

### 2.3. Extraction of Genomic DNA

Genomic DNA was extracted from Ficoll-separated peripheral blood mononuclear cell pellets, previously stored at −80 °C. Extraction was carried out with the specific QIA Amp DNA Mini Kit (Qiagen, Venlo, Netherlands) according to the protocol provided by the manufacturer. The DNA isolated from the column was eluted in 50 µL of sterile water and stored at −20 °C until use. The concentration and quality of the extracted DNA were analyzed with the Bio-Analyzer (Bio-Rad, Hercules, CA, USA) device.

### 2.4. Mutational Analysis of the MUC5B Variant rs35705950

The SNP variant rs35705950 of the MUC5B gene promoter has been sought in ccfDNA and genomic DNA samples for comparison. Real-time quantitative PCR with the TaqMan^®^ SNP Genotyping Assay was performed according to the manufacturer’s instructions. Briefly, 4.5 µL of extracted DNA was adopted in a total volume of 10 µL to perform molecular analysis on the QuantStudio™ 5 Real-Time PCR platform (Thermo Fisher Scientific, Waltham, MA, USA). Each sample, along with negative and positive control, was processed in duplicate and manually inspected using the Thermo Fisher ™ Cloud software (Thermo Fisher Scientific).

### 2.5. Statistical Analysis

Numerical variables were described using the mean ± standard deviation (SD) in the symmetrical distribution or the median with interquartile range [25th; 75th percentile] in case of variables showing consistent skewness. Categorical variables were summarized using absolute frequencies and percentages. The Kolmogorov–Smirnov test was used to assess normality. Accordingly, comparisons between IPF patients and healthy controls were based on the Mann–Whitney U test. Correlations among numerical variables were based on the non-parametric Spearman rank correlation coefficient. The median concentration of small length (100–300 bp) ccfDNA was used to group patients with low vs. high levels. Distribution analysis according to disease severity was analyzed by Fisher exact test. All tests were two-tailed. A *p*-value < 0.05 was considered significant. All statistical analyses were performed using the GraphPad Prism 7 (GraphPad Software Inc., San Diego, CA, USA) platform.

## 3. Results

### 3.1. Patient Characteristics

The study population included 59 therapy naïve and clinically stable IPF patients and 35 sex- and age-matched controls. Demographics and clinical data are shown in Table 1. Main comorbidities included systemic arterial hypertension (42%), gastro-esophageal reflux (34%), type II diabetes (24%), and ischemic cardiovascular disease (24%). Overall, respiratory function testing showed that patients had a mild to moderate restrictive ventilatory pattern with the same degree of DLCO_sb_ deficit. As shown, most patients had a mild to moderate illness, according to the GAP staging system. Moreover, TORVAN stages I–III were the most represented, with a minority of cases in stage IV (Table 1).

### 3.2. Levels of Circulating Cell-Free DNA Are Significantly Increased in IPF Patients

**Figure 1:**
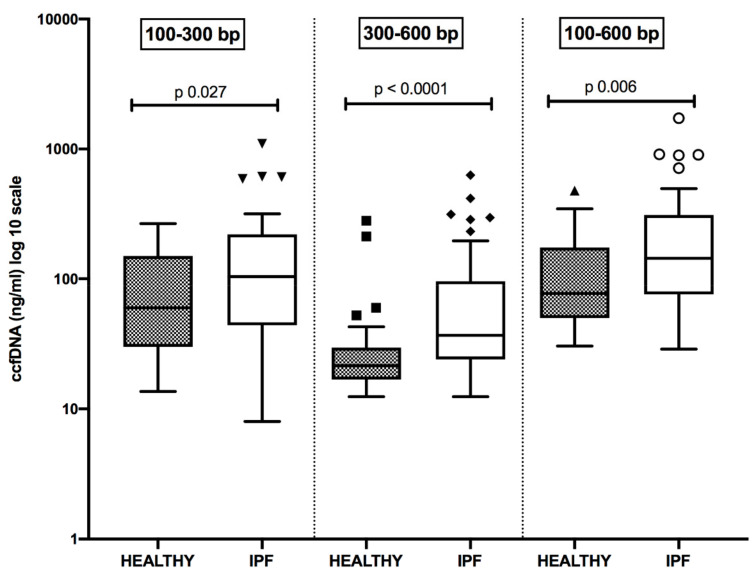
Distribution of plasma concentrations (log 10 scale) of small size circulating cell-free DNA (ccfDNA) in healthy subjects and patients suffering from idiopathic pulmonary fibrosis (IPF). All comparisons are statistically significant.

Figure 1 shows the concentration distribution of small-length ccfDNA, respectively represented by fragments of 100–300 bp and 300–600 bp, in plasma samples of newly diagnosed and therapy-naïve IPF patients and controls. The sum of fragments of different lengths is also reported. In all comparisons, ccfDNA levels were significantly increased in IPF patients than in control subjects. Moreover, the smallest fragments of 100–300 bp were more represented than those of 300–600 bp, both in controls (*p* = 0.0007) and in IPF cases (*p* < 0.0001).

Detailed data are reported in Table 2.

**Figure 2:**
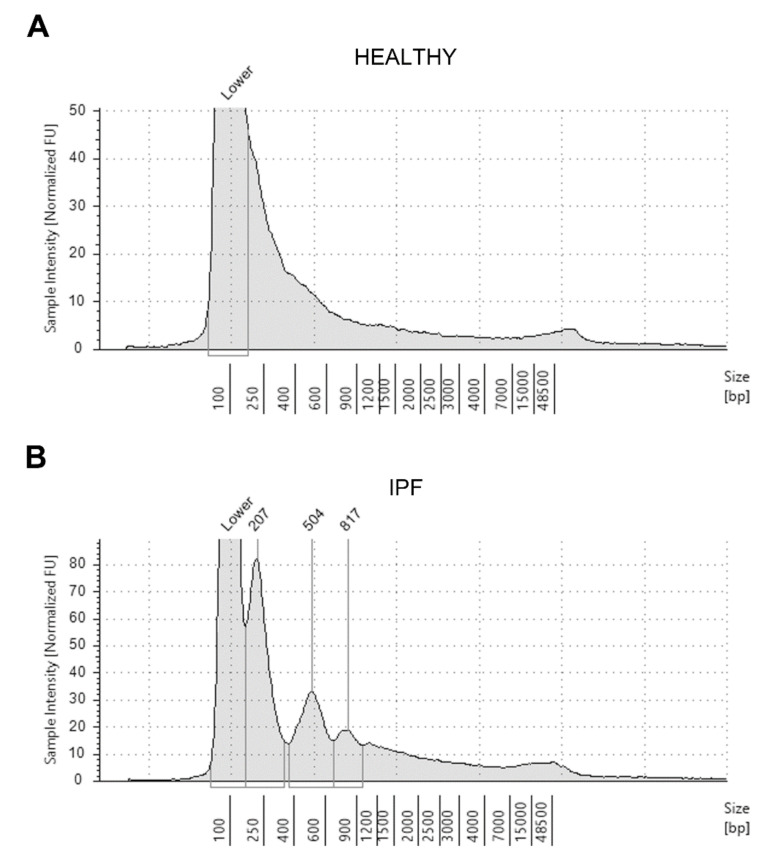
Representative electrophoretic run and distribution pattern of circulating cell-free DNA extracted from the plasma sample of an healthy individual (**A**) and a patient affected by idiopathic pulmonary fibrosis (IPF) (**B**), as analyzed with the Tape Station 2200 system (see Methods section).

Representative graphs that allow catching the differences in the distribution pattern of ccfDNA fragments according to their size in a healthy subject (panel A) and an IPF patient (panel B) are shown in Figure 2.

### 3.3. Levels of Circulating Cell-Free DNA Is Associated with Lung Function Decline and Disease Severity in IPF Patients

**Figure 3:**
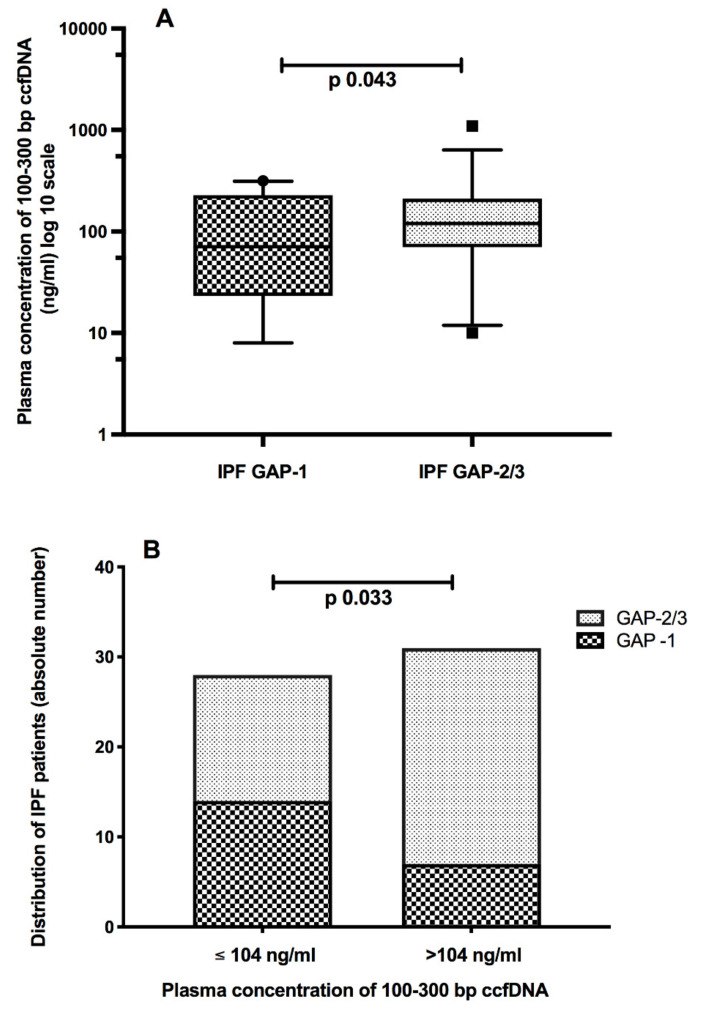
Plasma concentrations of 100–300 bp circulating cell-free DNA (ccfDNA) were significantly higher in idiopathic pulmonary fibrosis (IPF) patients with more advanced disease, according to the GAP (gender-age-physiology) staging score (**A**). The median cut-off of 104 ng/mL helps discriminating patients with advanced vs. milder disease (**B**).

As shown in Figure 3A, plasma expression of 100–300 bp ccfDNA fragments was significantly higher in GAP-2 and GAP-3 IPF patients than in cases with milder illness (GAP-1). The median value of 104 ng/mL allowed discriminating patients with mild disease (GAP-1) from those more advanced (GAP-2 and GAP-3) (Figure 3B). No differences were observed with the TORVAN staging (data not shown). Moreover, plasma levels of 100–300 bp ccfDNA fragments were significantly indirectly related to the DLCO_sb_ impairment (expressed as % of predicted), and the distance walked (meters) at the 6-MWT in IPF patients (Spearman *r* = −0.28, 95% CI −0.51 to 0.00, *p* = 0.03 and Spearman *r* = −0.32, 95% −0.57 to 0.00, *p* = 0.04, respectively). No significant data were detected concerning longer ccfDNA fragments (300–600 bp).

### 3.4. Circulating Cell-Free DNA Carries Genetic Information

To address whether ccfDNA may carry any genetic information, we analyzed the expression of the rs35705950 promoter polymorphism of the MUC5B gene. Paired genomic DNA was used for comparison. The rs35705950 polymorphism was found in the ccfDNA sample of 7 of 59 IPF patients (11.8%) and 3 of 35 controls (8%), with no differences. Genotype concordance between ccfDNA and genomic DNA was detected in all cases in the control group. Conversely, four out of 7 IPF ccfDNA samples carrying the rs35705950 polymorphism (57%) were discordant from genomic DNA, accounting for 6.7% of the whole IPF patient group. The head-to-head genotype comparison of ccfDNA vs. genomic DNA in the two study groups is reported in Table 3.

## 4. Discussion

The present study evaluated plasma concentrations of small length fragments of circulating cell-free DNA in a cohort of newly diagnosed and therapy naïve patients affected by IPF. Recently, much importance has been given to liquid biopsy as noninvasive techniques, based on samples from blood or other body fluids, may allow detecting materials, i.e., DNA, originating from the disease site [11,12,22,23,24]. Our effort represented a pioneering attempt to address whether there are the conditions for using liquid biopsy as a suitable tool in the diagnostic and prognostic disease processes. Casoni et al. first showed in 2010 that serum concentrations of circulating free DNA were higher in IPF patients than in healthy donors and patients suffering from other ILDs. Besides, circulating free DNA was suggested as a useful biomarker discriminating IPF from non-IPF cases [15]. Despite this optimistic expectation, further studies have not been carried out in the field. More than ten years later, our findings demonstrate that ccfDNA was readily detectable in both plasma samples of healthy subjects and IPF patients. As expected, and according to previous data in cancer patients [25,26], ccfDNA was higher in IPF cases than in healthy controls. Moreover, small-size (100–300 bp) ccfDNA fragments were significantly higher in patients in GAP-2 and GAP-3 stages than in GAP-1 cases, with a median cut-off of 104 ng/mL discriminating patients with more advanced vs. milder disease. This finding was further supported by the inverse relationship between higher levels of 100–300 bp ccfDNA fragments and lung function parameters, including the impairment of the CO lung diffusion capacity and the reduced distance walked. Cancer patients present higher levels of circulating DNA when the tumor burden increases [27]. High disease-related cell turnover helps explain the increased release of DNA fragments from dead cells into the bloodstream [28]. Likely, a similar process will also occur during IPF.

While our study aligns with that of Casoni et al. [15], some differences merit to be outlined. We have chosen to analyze plasma instead of serum samples to ensure a better yield in ccfDNA recovery. Moreover, plasma instead of serum-derived ccfDNA is a more suitable target for genetic analysis [29,30]. The use of advanced purification and quantification technologies has been an additional element of strength. Indeed, we have been able to show that the distribution pattern of ccfDNA fragments was different in the controls than in the patients, thanks to using a sort of nucleic acid run, which, at the same time, allowed us to highlight any contamination by genomic DNA. Detection of ccfDNA in control subjects should not surprise. Circulating cell-free fragments of 180–200 bp have been reported in tissue turn-over under physiological conditions due to DNA degradation related to apoptosis and necrosis events [31]. Moreover, some studies have shown that ccfDNA levels are even more increased in healthy people under stress or during physical activity [32,33].

The oncology setting is where liquid biopsy finds greater application. This because bloodstream cell-free DNA contains small percentages (<10%) of circulating tumor-derived DNA that carries disease-related genetic and epigenetic information [34]. It is widely recognized that the early use of this information has significantly improved both the knowledge and the clinical management of the oncological patient. For instance, in non-small cell lung cancer patients with no available tumor biopsy, blood is the only source for detecting genetic alterations, including EGFR, KRAS, BRAF, PIK3CA mutations, and ALK rearrangements [11,30]. Besides, in patients with specific genetic abnormalities at presentation, serial liquid biopsies allow monitoring treatment effectiveness through the early detection of any mutation-induced-drug resistance.

Idiopathic pulmonary fibrosis shares many similarities with cancer, both in pathogenesis and disease behavior [13]. The gold standard for IPF diagnosis relies on recognizing the UIP pattern on chest HRCT in the appropriate clinical context when alternative diagnoses have been carefully excluded. In the case of atypical HRCT presentation, lung biopsy is recommended [2]. However, not all patients are eligible for age and comorbidity limits. Having made these observations as a whole, the availability of easy-to-perform tools can be of help to simplify holistic disease management. According to this aim, we analyzed the expression of the rs35705950 promoter polymorphism of the MUC5B gene in ccfDNA samples of our study cohort. We have chosen this gene target, encoding for a major mucus component of the respiratory tract, as it is the most studied in the field of IPF. The MUC5B promoter polymorphism rs35705950 (T allele, either heterozygous or homozygous) is also the most frequently encountered gene alteration in sporadic IPF and familial pulmonary fibrosis. It is associated with increased susceptibility to developing the disease and a better prognosis [6,7,8,9]. To the best of our knowledge, this is the first report showing that liquid biopsy allows detecting this polymorphism in ccfDNA both in healthy controls and IPF patients, with no difference. This finding was not entirely surprising due to the not negligible prevalence of the polymorphism, estimated at around 10%, in the general population [35,36]. Very interestingly, the ccfDNA rs35705950 polymorphism was also present at the DNA genomic level in healthy controls, while this was not the case in IPF patients. Indeed, in four out of seven cases carrying the polymorphism, the ccfDNA finding was discordant from paired genomic DNA. In our opinion, this observation is of utmost relevance as it likely reflects the presence within the whole ccfDNA sample of DNA fragments directly released from the site of the disease in analogy with cancer biology. Given this hypothesis, the genetic information that these DNA fragments contain may be representing a somatic mutation acquired at the disease site instead of representing a germline signature. If this insight was confirmed in larger study populations, it would substantially impact disease management and open new research scenarios. Very likely, we believe that our finding may not be specific for IPF but also expected in other fibrotic ILDs. Future studies exploring this possibility through a comparison of blood vs. lung tissue samples representative of the disease site should be encouraged. 

We are aware that our study has several limitations. First of all, it is a single-center study with a small size population. Despite these limits and the retrospective nature, the operating yield of our research has, however, been sufficient to pursue the objectives set. Plasma processing is a critical issue in ccfDNA analysis. Pre-analytical procedures (i.e., time frame for sample processing following collection to avoid lysis of blood cells) remain to be standardized. Prospective and larger efforts are certainly needed to confirm our explorative data and establish reproducible and reliable technical protocols. Finally, investigating additional genetic targets may help improve early diagnosis, tailor therapy strategies, and stratify patients for prognostication purposes. 

## 5. Conclusions

In conclusion, our data show that plasma ccfDNA concentrations were high in IPF patients, increased in more advanced disease, and were negatively correlated with lung function. Bloodstream cfDNA carried the germline rs35705950 MUC5B polymorphism, and this finding was fully concordant with genomic DNA in controls. Hopefully, the observation of discrepant cases between ccfDNA and genomic DNA in IPF patients suggests that the former may convey specific genetic information present in the primary site of the disease.

## Figures and Tables

**Table 1:** Demographics and clinical characteristics of the study population.

Parameter	Healthy (*n* = 35)	IPF (*n* = 59)	*p*
Gender, M (%)	23 (65)	42 (71)	ns
Age (years)	67.5 ± 5.9	69.4 ± 9.2	ns
Smoking status (current/former/never smoker)	4(11)/24(69)/7(20)	0(0)/43(73)/16(27)	ns
Forced vital capacity (% pred)	-	72.0 [56.7–84.2]	
Total lung capacity (% pred)	-	56.5 [46.0–72.0]	
DLCO_sb_ (% pred)	-	45.0 [35.0–63.0]	
6-MWT distance (m)	-	392.0 [300–517.5]	
GAP stage (I/II/III)	na	21(35.5)/28(47.5)/10(17)	
TORVAN (I/II/III/IV)	na	18(30)/14(24)/20(34)/7(12)	

Data are expressed as absolute number (%), mean ±SD or median [25th–75th], where appropriate. Abbreviations: IPF = idiopathic pulmonary fibrosis; M = males; DLCO_sb_ = single breath diffusion lung capacity for carbon monoxide; 6-MWT = 6-minute walk test; m = meters; GAP = gender-age-physiology; na = not applicable; ns = not significant.

**Table 2:** Plasma concentrations of different size ccfDNA in healthy and IPF patients.

Parameter (ng/mL)	Healthy	IPF	*p*
100–300 bp	60.0 [30.0; 150.0]	104.0 [44.0; 220.0]	**0.0276**
300–600 bp	21.48 [16.80; 29.60]	36.80 [24.0; 96.0]	**<0.0001**
100–600 bp	77.20 [50.0; 174.0]	144.0 [76.0; 310.0]	**0.0061**

Data are expressed as or median [25th–75th]. Statistically significant results are reported in bold. Abbreviations: IPF = idiopathic pulmonary fibrosis; bp = base pair.

**Table 3:** Distribution of the rs35705950 MUC5B polymorphism in circulating cell-free DNA and genomic DNA for comparison in healthy and IPF patients.

Healthy			
**ccfDNA**	**Genomic DNA**		
	G/G	G/T	T/T
G	32	0	0
T	0	0	3
G + T	0	0	0
**IPF**			
**ccfDNA**	**Genomic DNA**		
	G/G	G/T	T/T
G	49	3	0
T	4	0	3
G + T	0	0	0

Abbreviations: IPF = idiopathic pulmonary fibrosis; ccfDNA = circulating cell-free DNA.

## Data Availability

All data of interest were collected anonymously from clinical charts into a dedicated database.
